# Perceptions of ostrich welfare and ethical practices: insights from consumers, farmers and stakeholders

**DOI:** 10.1007/s11250-026-05422-x

**Published:** 2026-10-20

**Authors:** Maud Bonato, Monique Dawson, Anel Engelbrecht, Schalk W.P. Cloete

**Affiliations:** 1 Department of Biological Sciences, University of Venda, Private Bag X5050, Thohoyandou, 0950 South Africa; 2 Department of Animal Sciences, University of Stellenbosch, Matieland, 7602 South Africa; 3 Directorate Animal Sciences, Western Cape Department of Agriculture, Oudtshoorn, 6620 South Africa

**Keywords:** Animal husbandry, Ethical standards, Perception differences, Survey, *Struthio camelus*

## Abstract

This study investigated South African consumers’, farmers’, and industry stakeholders’ perceptions of ostrich welfare using a questionnaire distributed nationwide between September 2018 and September 2019. The survey included demographic questions and assessed knowledge of ostrich husbandry, welfare priorities, and purchasing behaviors. A total of 302 participants completed the questionnaire. A Principal Component Analysis identified three principal components that explained 49.5% of the total variance: ostrich welfare priorities, knowledge of ostrich production and management, and health and ethical considerations in ostrich farming. Welfare priorities and knowledge levels were influenced by demographic factors, with farmers consistently scoring the highest on knowledge and consumers the lowest. All participants reported a modest understanding of husbandry practices but placed a high importance on welfare-related factors such as feeding, housing, health, and minimizing pain and stress. Although most participants preferred welfare-conscious products, their willingness to pay a premium was low. These findings highlight a strong welfare orientation across groups but reveal knowledge gaps and an attitude–behavior gap, suggesting opportunities for targeted education, improved industry awareness and transparency, and welfare-focused product communication.

**Supplementary Information:**

The online version contains supplementary material available at https://doi.org/10.1007/s11250-026-05422-x.

## Introduction

Over the past 50 years, the concept of animal welfare has shifted from an emphasis on basic physical health and functioning to a more comprehensive understanding that includes mental health and the expression of natural behaviors (Broom 2011). The Brambell Report (Brambell 1965) laid the foundation for modern farm animal welfare by recommending that animals should have sufficient freedom to stand up, lie down, turn around, groom themselves and stretch their limbs. These principles were subsequently developed into the “Five Freedoms” by the Farm Animal Welfare Council (FAWC, 1979), which emphasized freedom from hunger and thirst, discomfort, pain, injury and disease, fear and distress, and the freedom to express normal behavior. Welfare science further expanded in the 1990s with David Fraser’s “Three circles” framework, which highlighted animals’ affective states, biological functioning and natural living (Fraser 1999; Mellor and Beausoleil 2015). In addition, technological advances, such as automated monitoring systems, enable welfare assessments in agricultural and laboratory settings (Broom 2011). By enhancing human ability to observe and interpret animal behavior, these technological advances also contributed to a deeper understanding of animal emotional intelligence and cognition, aligning with the increasing societal expectations for more ethical and humane treatment of production animals (Mellor and Beausoleil 2015).

This concept of animal sentience is, however, rarely agreed upon by all parties and has, as such, sparked increased interest worldwide (Hewson 2003). As the demand for animal products continues to rise, so does the scrutiny of how these animals are raised and treated. This conflict between the need for more efficient production systems to meet growing food security demands and the call for humane animal treatment often raises public concerns about the ethical implications of intensive farming practices. In this regard, several studies have shown that consumers are becoming increasingly aware of these issues and are therefore also increasingly advocating for better welfare standards for farmed animals (Alonso et al. 2020; Prickett et al., 2010). Moreover, this has prompted various stakeholders, including consumers, NGOs, and policymakers, to call for greater transparency in the livestock production sector. As a result, more producers are adopting higher welfare standards to meet consumer expectations, reflecting a market shift towards more ethically produced products (Chen et al. 2024). Consequently, the intersection of animal welfare and food production systems is becoming a critical area of discussion, influencing both consumer choices and industry practices.

Generally, the public perceives intensive animal production systems used to meet the ever-growing nutritional demands of the population as compromising animal well-being (Henchion and Zimmermann 2021). Issues related to health, management, facilities, equipment, behavior and distress are typically thought to be associated with these intensified production systems (Čobanović and Magrin 2023). Both consumers and non-consumers of animal products are increasingly concerned about the welfare of farmed animals, alongside food safety and the environmental consequences of food production systems (Broom 2011; Alonso et al. 2020). Understanding how the public perceives animal welfare is therefore vital, as animal welfare is a social construct that reflects society’s values (Sinclair et al. 2022). This is because societal values and norms shape public behavior, which in turn influence buying decisions. One challenge is that the widening distance between consumers and farmers influences consumer perceptions due to a general lack of knowledge. Warsaw et al. (2021) demonstrated that most modern-day consumers have no direct contact with the farms that produce the food they purchase (Bouwman et al. 2024; Hasanzade et al. 2022). This physical disconnect between consumers and farmers has also widened the mental distance between the two groups, thereby increasing the differences in their common morality (Bouwman et al. 2024). This can be directly related to globalization and the increasing influence of social media on creating a narrative that is not necessarily based on facts but appeals to emotions and people’s ignorance.

The ostrich industry is a niche industry that primarily sells its products to luxury markets, far removed from the farms where the birds are farmed. South Africa, a major global producer and exporter of ostrich commodities, plays a crucial role in international markets, with up to 90% of its products exported to the European Union (DALRRD, 2021). However, while European consumers prefer higher-quality products, they also increasingly demand sustainable and ethical production practices, traceability, and product integrity (Gao and Wei 2023), placing additional strain on the successful marketing of ostrich products. Ostrich farming practices are not widely known in most parts of the world, and the lack of knowledge could therefore influence consumers’ buying decisions, particularly among the growing number of people who prefer ethically produced products (Yue et al. 2020), in a world where animal-derived products are increasingly questioned. Ostriches are officially categorized as poultry (DoA, 2006; WOAH, 2025). This classification risks creating a misleading association with conventional poultry farming methods (such as high-density stocking, intensive production, and cage confinement), which may generate an inaccurate perception of how ostriches are actually raised.

Against this background, this study used a questionnaire to assess and compare public and stakeholder knowledge and perceptions of the South African ostrich industry. Specifically, it sought to gain a comprehensive understanding of the differences in viewpoints between these groups in South Africa regarding (1) their knowledge of current production processes used in the industry, (2) their perceptions of the welfare implications associated with ostrich production, (3) their purchasing behavior and (4) their preferences regarding ostrich husbandry practices. We hypothesized that the ostrich farming industry is relatively unfamiliar to most South Africans compared to other livestock industries and that participants’ knowledge of the industry might affect their opinions and perceptions regarding the welfare of farmed ostriches.

## Materials and methods

### Study description and design

A customized questionnaire was distributed nationwide from September 2018 to September 2019 to assess perceptions and opinions of farmed ostriches in South Africa among consumers, commercial ostrich farmers, and secondary stakeholders (i.e. veterinarians, nutritionists, and processors). Available in both Afrikaans and English, the questionnaire aimed to reach a diverse range of socio-demographic and lifestyle backgrounds. An online survey platform (Google Forms^®^) was used to facilitate nationwide distribution of the questionnaire. The online questionnaire was distributed via email to the South African Society of Animal Science (SASAS), the Animal Feed Manufacturers Association (AFMA), the Western Cape Department of Agriculture and the University of Stellenbosch. Physical copies were also distributed in Oudtshoorn, the “ostrich capital of the world” in the Western Cape region of South Africa, where the largest global population of farmed ostriches is found. Ostrich farmers were also specifically targeted at farmers’ meetings in key ostrich-farming areas of the Klein Karoo and Southern Cape, including Herold, De Rust, Volmoed, and Heidelberg.

### Design of the questionnaire and measurement scale

A multidisciplinary approach was used to develop the questionnaire, incorporating resource- and management-based welfare measures alongside animal-based outcomes. The four principles of the Welfare Quality approach (i.e. good feeding, good housing, good health, and appropriate behavior) served as the foundation for the questionnaire (Botreau et al. 2009). Additionally, the topics included were informed by literature reviews of welfare indicators and measurements commonly used in other surveys to gauge public opinions and preferences regarding animal welfare. The questionnaire also addressed topics on consumer acceptance and preferences, focusing on both on-farm and end-product standards (Vanhonacker et al. 2008; Bejaei and Cheng 2014; You et al. 2014; Miranda-de la Lama et al., 2017). Responses to the questionnaire were recorded using five-point Likert scales. (Miranda-de la Lama et al., 2017). Scores were allocated from least to most knowledgeable/important/likely. Although Likert-scale data are ordinal in nature, the five-point response scales were commonly treated as approximately continuous for exploratory multivariate analyses, as the primary objective was to identify patterns and reduce dimensionality, rather than to undertake psychometric validation.

The survey was divided into two sections. The first section (Section A, Appendix 1) focused on collecting demographic information to assess the socio-demographic characteristics of the sampled population. This section included questions on age, gender, occupation, education level, income, province of residence, and dietary preferences to assess how these factors influenced attitudes toward ostrich welfare. The second section (Section B, Appendix 1) focused on participants’ general views on commercial ostrich production, their knowledge of ostrich husbandry practices, their perceptions of ostrich welfare, and how these factors influence their purchasing decisions.

### Data preparation and pre-processing

All data preparation/cleaning and analysis were performed in R (Core Team 2025). The original dataset included all responses assessing participants’ understanding, attitudes, and behaviors related to ostrich production and management (Questions 1a to 6b: Appendix 1). Prior to statistical analyses, the dataset was examined for completeness and missing values using the *dplyr* package (Wickham et al. 2023). Of the possible 12 080 questionnaire responses, 148 (1.22%) were missing. Missing responses were dispersed across questionnaire items, and no individual variable contained a substantial proportion of missing observations (Supplementary Table S2). Given the low overall proportion of missing data, all respondents were retained in the analysis by inputting missing values using the regularized Principal Component Analysis (PCA) algorithm implemented in the *missMDA* package (Josse and Husson 2016). The optimal number of Principal Components (PCs) for the imputation procedure was estimated using *estim_ncpPCA*, and missing values were subsequently imputed using *imputePCA* (Josse and Husson 2012, 2016). To evaluate the influence of missing-data imputation on the PCA results, a sensitivity analysis was conducted using *FactoMineR* (Lê et al. 2008) by repeating the principal component analysis on complete items only. The component structure obtained from the complete-case analysis was then compared with that from the imputed dataset by examining the retained components, eigenvalues, variance explained, and variable-loading patterns.

### Principal component analysis (PCA)

Prior to conducting the PCA, the suitability of the questionnaire data for factor analysis was assessed using the *psych* package of R (Revelle 2026), with the Kaiser-Meyer-Olkin (KMO) measure of sampling adequacy and Bartlett’s test of sphericity. The KMO statistic was 0.91, indicating excellent sampling adequacy. Bartlett’s test of sphericity was highly significant (χ² = 8365.17, df = 780, *p* < 0.001), confirming that the correlation matrix was appropriate for PCA. PCA was subsequently performed using Pearson correlations using the *FactoMineR* package (Lê et al. 2008). This approach has been shown to yield robust results for Likert-scale data with five or more response categories and moderate-to-large sample sizes (Norman 2010; Rhemtulla et al. 2012). PCA was thus used as a data-reduction technique to summarise correlated questionnaire items into a smaller number of orthogonal PCs representing the major patterns of variation in the participants’ responses. The analysis was performed without rotation, as the primary objective was data reduction to summarize the questionnaire responses into a smaller number of components for subsequent analyses, rather than identifying latent constructs. The percentage of explained variance, a scree plot, and Horn’s parallel analysis were used to determine the number of meaningful principal components to retain for further analysis and interpretation, using the *ggplot2* and *psych* packages. In addition, an internal reliability test (measured by Cronbach’s α) was assessed using the *psych* package. Finally, a sensitivity analysis comparing the PCA performed on the imputed dataset with a complete-case PCA was conducted to assess the influence of the imputation procedure on the retained principal components using the *factoextra* and *ggplot2* packages (Kassambara et al. 2026; Wickham et al. 2024).

### Statistical analysis

To explore whether participants’ PC scores differed according to demographic or behavioral characteristics, mean factor scores were compared across various categorical predictors, including category (consumers, farmers or stakeholders), gender, education, income, diet, and province of residence. Normality of factor score distributions was assessed using the Shapiro–Wilk test. As the assumptions of normality were not met, a Kruskal–Wallis test was used uniformly, regardless of the number of groups. Furthermore, as multiple demographic comparisons were conducted, p-values obtained from the Kruskal-Wallis tests were adjusted using the Benjamini-Hochberg false discovery rate procedure to reduce the risk of Type I errors. For each significant factor (*p* < 0.05), mean scores and standard errors were calculated for each level of these categorical predictors. Negative scores indicated values below the dataset mean, while positive scores indicated values above the mean. When a significant overall effect was detected, pairwise comparisons were performed using Dunn’s post hoc test with Bonferroni-adjusted p-values in the *FSA* package (dunnTest function) to control for multiple comparisons (Ogle et al. 2026). In addition, effect sizes were calculated using the rank-based eta-squared (η²[H]) statistic, implemented in the *rstatix* package (Kassambara 2019). This value was calculated as η²[H] = (H − k + 1)/(n − k), where H is the Kruskal-Wallis statistic, k the number of groups, and n the total sample size. Because η²[H] is a bias-corrected estimator, it can take slightly negative values when the observed H is smaller than expected under the null; such values are interpreted as no effect (Tomczak and Tomczak 2014).

## Results

### Data description: socio-demographic profile of the participants

A total of 302 South African participants completed and returned the questionnaire, comprising 175 (58.0%) consumers, 96 (31.8%) secondary stakeholders in the ostrich industry (i.e. processors, tanners, veterinarians and other industry representatives), and 31 (10.3%) ostrich farmers. Gender information was available for 298 respondents, of whom 193 (64.8%) identified as male and 105 (35.2%) as female.

Age information was available for 297 respondents, all of whom were older than 20 years. For analyses, participants were grouped into three age categories: younger than 36 years, 36–50 years, and older than 50 years (Table 1), with 143 (48.1%) that were younger than 36 years, 90 (30.3%) were between 36 and 50 years, and 64 (21.6%) were older than 50 years.

**Table 1:**
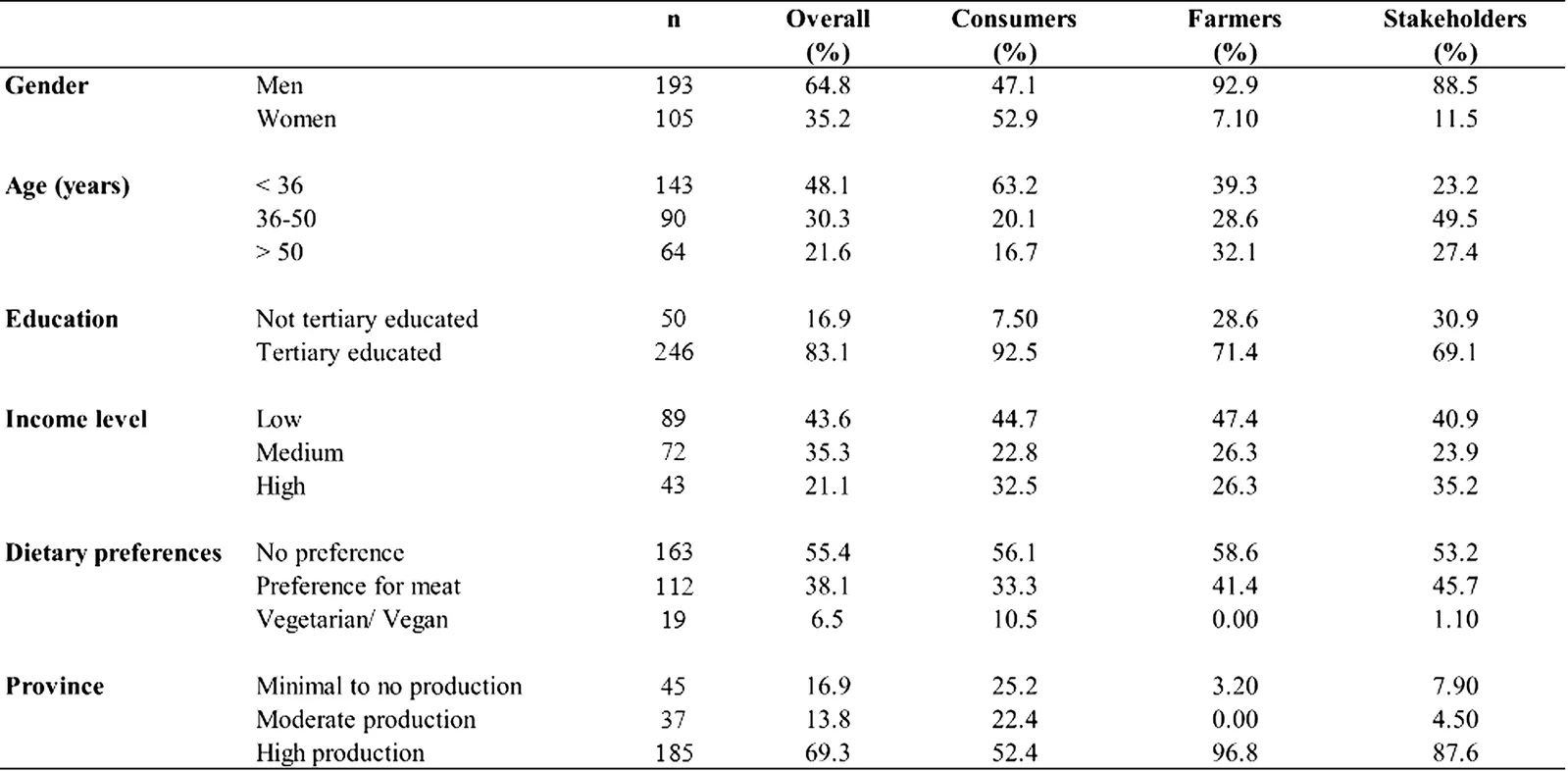
Demographic characteristics of the questionnaire study participants overall and within participant categories (consumers, farmers, and stakeholders). Participants’ income levels were defined as follows: low-income (below R200 000 per year), medium-income (between R200 000 and R600 000 per year), and high-income (above R600 000 per year). Province of residence was defined as follows: minimal to no ostrich production (KwaZulu-Natal, Gauteng, Mpumalanga, North West and Free State); moderate ostrich production (Eastern Cape, Northern Cape and Limpopo); and province with high ostrich production (Western Cape)

Note: n = number of respondents. Overall percentages are based on respondents providing valid information for each demographic variable. Percentages for consumers, farmers, and stakeholders are calculated within each participant category; therefore, they sum to 100% within each category

Education level was reported by 296 respondents. Following regrouping for statistical analyses, 246 respondents (83.1%) were classified as having tertiary education (college or university diploma/degree), while 50 respondents (16.9%) were classified as not having tertiary education (primary and secondary education combined; Table 1).

Income information was provided by 204 respondents. Of these, 89 respondents (43.6%) were classified as low income (below R200 000 per year), 72 (35.3%) as medium income (R200 000–R600 000 per year), and 43 (21.1%) as high income (above R600 000 per year; Table 1).

The province of residence was reported by 267 respondents. Of these, 45 (16.9%) resided in provinces with minimal or no ostrich production (KwaZulu-Natal, Gauteng, Mpumalanga, North West and Free State), 37 (13.8%) in provinces with moderate ostrich production (Eastern Cape, Northern Cape and Limpopo), and 185 (69.3%) resided in the province with the highest ostrich production (Western Cape; Table 1).

Finally, dietary preference was reported by 294 respondents. Of these, 163 (55.4%) indicated no specific dietary preference, 112 (38.1%) reported consuming meat, and 19 (6.5%) identified as either vegetarian or vegan, which were combined into a single category for analyses owing to the relatively small numbers in each group (Table 1).

### General trends in the sampled population

Overall, participants indicated limited to average knowledge of the husbandry practices used in the ostrich industry, with mean (± SEM) scores ranging from 2.41 ± 0.07 to 2.85 ± 0.07 (Table 2). Nevertheless, relatively high scores (4.11 ± 0.05 to 4.71 ± 0.03) were attributed to the welfare importance of basic feed, water, housing, and health needs for the commercial production of ostriches, as well as to the importance of limiting stress and pain (mean scores of 4.39 ± 0.04 and 4.42 ± 0.05, respectively). Although slightly lower scores were observed compared to the latter, participants tended to consider factors related to natural living (e.g. the ability to exhibit natural behavior, freedom of movement and environmental enrichment), relatively important with mean scores ranging from 4.00 ± 0.05 to 4.23 ± 0.05 (Table 2). Regarding management and production practices, participants assigned relatively high importance to the skill level of stockmen, isolation of sick/injured birds, frequent visual inspection, type of vehicle used for transportation, and use of preventive medicine, with mean scores ranging from 4.24 ± 0.05 to 4.55 ± 0.04 (Table 2). Furthermore, participants generally attributed relatively high importance to stockmen’s knowledge of all factors proposed (i.e., behavior, detecting signs of stress and illness, handling/restraining and transporting of ostriches; with scores between 4.33 ± 0.04 and 4.68 ± 0.03; Table 2). Conversely, the importance of stockmen’s knowledge of the anatomy of ostriches, manually assisting chicks to hatch, and human rearing of chicks, received slightly lower scores of 3.93 ± 0.03, 3.39 ± 0.06 and 3.31 ± 0.06, respectively.

**Table 2:**
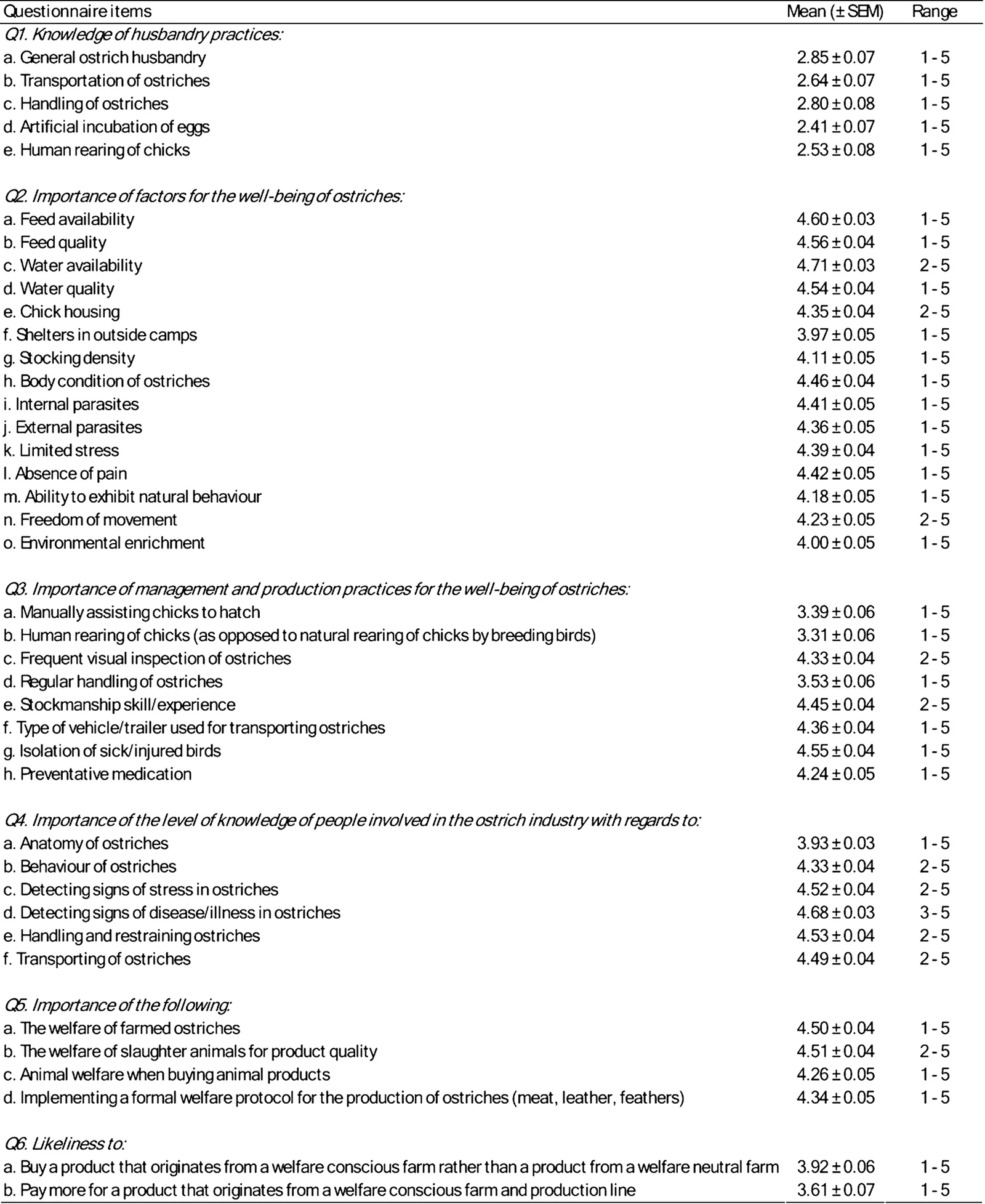
Mean scores (with standard errors) given by questionnaire participants regarding their knowledge of ostrich production, welfare perceptions, the importance of husbandry practices, and how welfare aspects affect their purchasing decisions. Scores are on a 1 to 5 scale, from least to most knowledgeable, important, or likely. Responses covered the full range of the scale for most dependent variables, indicating adequate variability in participant ratings

Finally, all participants considered the overall welfare of ostriches and the welfare of slaughtered animals, with respect to product quality, to be highly important (4.50 ± 0.04 and 4.51 ± 0.04, respectively). Similarly, they valued animal welfare when buying animal products and the implementation of a formal welfare protocol for the commercial production of ostriches as relatively important (4.26 ± 0.05 and 4.34 ± 0.05, respectively). However, while participants preferred purchasing welfare-conscious products over welfare-neutral ones (3.92 ± 0.06), they were more hesitant to pay a premium for them (3.61 ± 0.06).

### Principal component analysis

**Fig. 1:**
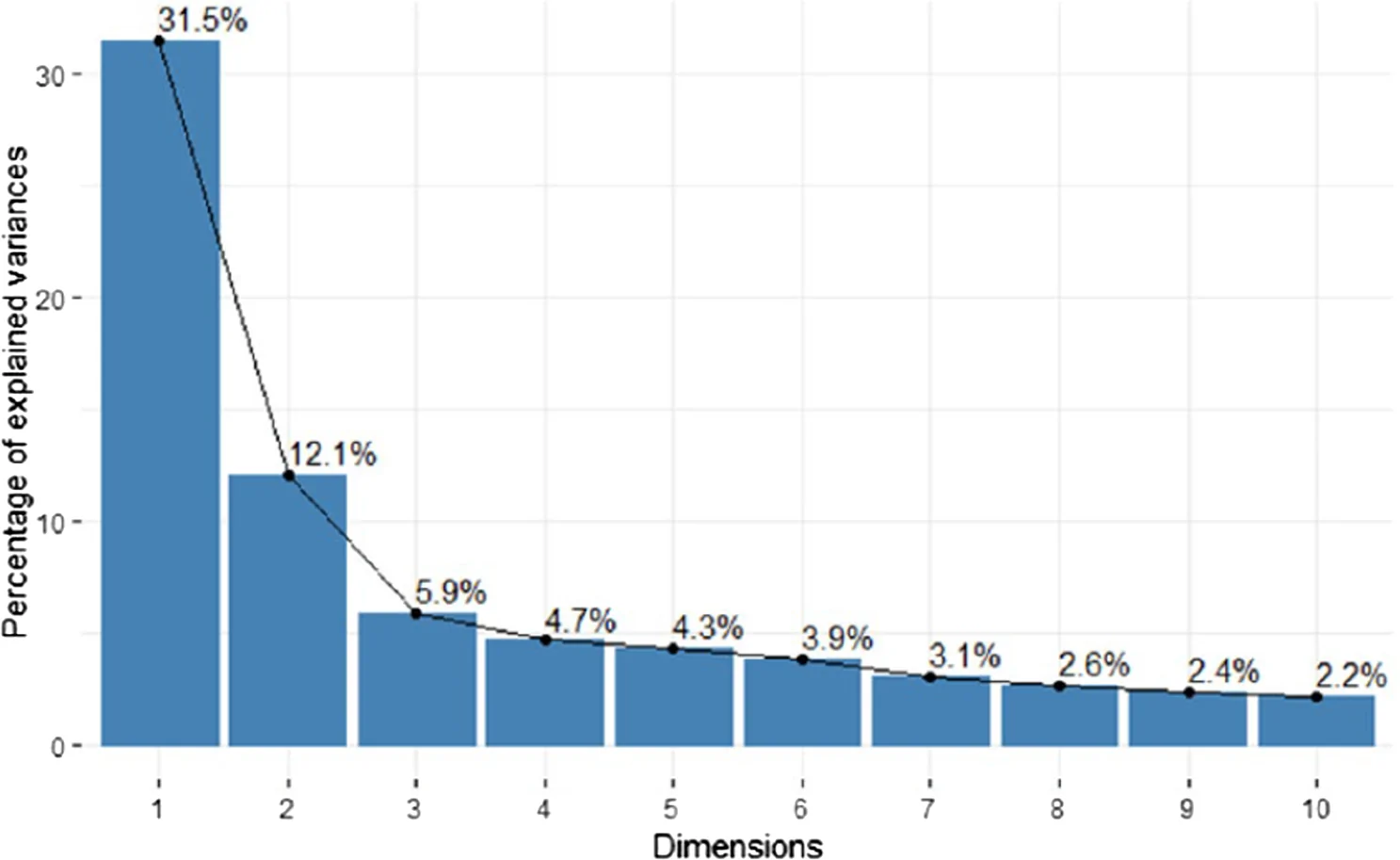
Principal component analysis (PCA) of the questionnaire responses showing the proportion of variance explained by each component. The first three principal components account for 49.5% of the total variance (PC1 = 31.5%, PC2 = 12.1%, PC3 = 5.9%), after which an evident elbow indicates diminishing returns from additional components

The PCA revealed that the first three components explained 49.5% of the total variance, while the subsequent components each explained progressively smaller proportions of the variance. Specifically, the first component (PC1) had an eigenvalue of 12.61 and accounted for 31.5% of the variance; the second component (PC2) had an eigenvalue of 4.83 and accounted for 12.08% of the variance; and the third component (PC3) had an eigenvalue of 2.37 and accounted for 5.93% of the variance. These levels are not uncommon for multidimensional attitudinal questionnaires in the behavioral and social sciences, where responses are influenced by numerous psychological and contextual factors (Hair et al. 2019; Tabachnick and Fidell 2019). The number of retained components was initially assessed using visual inspection of the scree plot (Fig. 1). Although the parallel analysis suggested retaining five components, only the first three principal components yielded coherent, interpretable PCs consistent with the study’s objectives. Furthermore, the component structure obtained from the complete-case analysis was highly consistent with that obtained from the imputed dataset. Correlations between the loading vectors of the imputed and complete-case PCA solutions were 0.990, 0.997 and 0.956 for the first three principal components, respectively, indicating that the low proportion of missing data (1.22%) and the imputation procedure had a negligible influence on the interpretation of the retained principal components (Supplementary Tables S3 and S4). Subsequently, three principal components were retained and assigned interpretive labels based on the questionnaire items with the highest loadings. PC1 (ostrich welfare priorities) was most strongly associated with questionnaire items on limited stress (Q2k), the welfare of farmed ostriches (Q5a), and knowledge of ostrich transportation (Q4f; Table 2). PC2 (knowledge of ostrich production and management) was most strongly associated with questionnaire items related to handling of ostriches (Q1c), general ostrich production (Q1a), and human rearing of chicks (Q1e), while PC3 (health and ethical considerations in ostrich farming) was strongly associated with questionnaire items on the importance of internal parasites for ostrich well-being (Q2i), the importance of external parasites for ostrich well-being (Q2j), and willingness to pay more for products originating from a welfare-conscious farm and production line (Q6b). The complete loading matrix for all questionnaire items is provided in Supplementary Table S1. The retained PCs demonstrated good internal consistency, with Cronbach’s alpha estimated at 0.75 for PC1, 0.94 for PC2 and 0.72 for PC3, indicating satisfactory reliability of the questionnaire items within each PC.

### Effect of demographic factors on principal components

Mean (± SEM) scores for ostrich welfare priorities (PC1) differed significantly by gender (χ² = 14.91, df = 1, η²[H] = 0.047, *adjusted p* = 0.0008) and province (χ² = 8.40, df = 2, η²[H] = 0.024, *adjusted p* = 0.04). Men had lower scores (–0.61 ± 0.28) compared to women (1.14 ± 0.26). Participants from the province with the highest ostrich production scored lower (–0.52 ± 0.27) than those from provinces with moderate ostrich production (0.96 ± 0.56; Z = -2.50, *p* = 0.03). No significant differences were observed between provinces with high and minimal to no ostrich production (0.57 ± 0.46; Z = -1.54, *p* = 0.37), or between provinces with moderate and minimal to no ostrich production (Z = -1.03, *p* = 0.90), indicating that the only significant difference was between provinces with high and moderate ostrich production. However, no significant differences were observed for category (χ² = 3.41, df = 2, η²[H] = 0.005, *adjusted p* = 0.42), income (χ² = 6.27, df = 2, η²[H] = 0.021, *p* = 0.13), education (χ² = 0.004, df = 1, η²[H] = -0.003, *p* = 0.95), or diet preference (χ² = 6.99, df = 3, η²[H] = 0.014, *p* = 0.19).

Scores for knowledge of ostrich production and management (PC2) differed significantly across all demographic factors, except income level (χ² = 0.96, df = 2, η²[H] = -0.005, *adjusted p* = 0.92). More specifically, significant differences were observed for category (χ² = 92.77, df = 2, η²[H] = 0.304, *adjusted p* < 0.0001), gender (χ² = 35.48, df = 1, η²[H] = 0.116, *adjusted p* < 0.0001), education (χ² = 11.72, df = 1, η²[H] = 0.036, *adjusted p* = 0.003), diet (χ² = 17.05, df = 3, η²[H] = 0.048, *adjusted p* = 0.003), and province (χ² = 26.16, df = 2, η²[H] = 0.092, *adjusted p* < 0.0001). Farmers scored highest (2.38 ± 0.25), followed by stakeholders (1.04 ± 0.19), while consumers scored lowest (–0.99 ± 0.14). Specifically, all three groups differed significantly from one another: farmers scored significantly higher than both stakeholders (Z = 2.86, *p* = 0.013) and consumers (Z = -7.79, *p* < 0.001), and stakeholders scored significantly higher than consumers (Z = -7.30, *p* < 0.001). Men had higher scores (0.54 ± 0.15) than women (–1.05 ± 0.21), and participants without tertiary education had higher scores (0.93 ± 0.28) than those with tertiary education (–0.24 ± 0.14). Participants with a preference for meat scored significantly higher than vegetarians (0.35 ± 0.20 and − 2.05 ± 0.53, respectively; Z = 4.09, *p* < 0.001), while participants with no dietary preference (–0.06 ± 0.17) also scored significantly higher than vegetarians (Z = 3.39, *p* = 0.004). No significant difference was observed between participants with a preference for meat and those with no dietary preference (Z = 1.62, *p* = 0.632). Finally, participants from provinces with the highest ostrich production scored significantly higher than those from provinces with minimal to no ostrich production (0.49 ± 0.16 and–0.83 ± 0.27, respectively; Z = 3.68, *p* < 0.001) and those from provinces with moderate ostrich production (0.49 ± 0.16 and − 1.14 ± 0.35, respectively; Z = 4.16, *p* < 0.001). No significant difference was observed between provinces with minimal to no ostrich production and those with moderate ostrich production (Z = 0.62, *p* = 1.000).

With regards to scores for health and ethical considerations (PC3), no significant differences were detected across any demographic factors: category (χ² = 2.02, df = 2, η²[H] = 0.001, *adjusted p* = 0.68), gender (χ² = 0.59, df = 1, η²[H] = -0.001, *adjusted p* = 0.73), education (χ² = 0.01, df = 1, η²[H] = -0.003, *adjusted p* = 0.94), income level (χ² = 1.60, df = 2, η²[H] = -0.002, *adjusted p* = 0.73), diet (χ² = 0.56, df = 3, η²[H] = -0.008, *adjusted p* = 0.94), or province (χ² = 0.69, df = 2, η²[H] = -0.005, *adjusted p* = 0.93).

## Discussion

The present study revealed several meaningful insights into the knowledge, attitudes, and priorities of South African citizens regarding welfare in the ostrich industry. The principal component analysis identified three main underlying PCs: ostrich welfare priorities, knowledge of ostrich production and management, and health and ethical considerations in ostrich farming, which varied across several demographic characteristics. Although the retained components only explained 49.5% of the total variance, this is well within the range commonly reported for exploratory analyses of behavioral and attitudinal questionnaire data, where responses are influenced by multiple underlying factors (Hair et al. 2019; Tabachnick and Fidell 2019). First, the participants’ relatively modest perceived knowledge of ostrich husbandry (mean scores between 2.4 and 2.9) contrasted with the high importance they assigned to animal welfare factors (e.g. feed, water, housing, health, and freedom from pain), with mean scores ranging from 4.1 to 4.7. Second, these PCs varied across demographic groups. Third, willingness to pay a premium for welfare-conscious products was comparatively lower (mean of 3.61) than the high importance placed on welfare itself.

The finding that participants mostly reported low to moderate knowledge of husbandry practices, yet great concern for welfare, is consistent with the broader body of research on consumer/stakeholder perceptions of farm-animal welfare. Several studies have reported that consumers often have limited factual knowledge of husbandry practices, production systems, or farm animal welfare conditions, yet they express strong sentiments about the welfare of farmed animals (Alonso et al. 2020; Fonseca and Sanchez-Sabate 2022; Sweeney et al. 2022). For example, Fonseca and Sanchez-Sabate (2022) found that consumers report low awareness of specific farming practices (e.g. tie stalls, one-day-old chick culling) but show higher levels of concern once these practices are made salient. In the present study, even stakeholders (many of whom likely have closer ties to production) indicated perceived gaps in their knowledge of husbandry practices in the ostrich industry. This has potential implications for education, extension and communication within the industry.

The strong emphasis placed on welfare-related factors (e.g., natural living, freedom of movement, enrichment, skilled stockmanship, and health or illness detection) suggests a growing welfare consciousness within agricultural production systems. Specifically, consumers worldwide increasingly expect ethical treatment of animals and view animal welfare as an integral part of food production. For example, Alonso et al. (2020) and Sweeney et al. (2022) showed that European consumers associate animal-friendly production with higher product quality, better health and safety credentials, and greater authenticity. In the South African context, a consumer study recently found that 84% of participants considered animal wellness very important when selecting animal products (NSF, 2024). Likewise, the ethical PC (health and ethical considerations) of the present study was uniformly high across demographics, reinforcing a baseline consensus or normative orientation towards welfare and ethics across all stakeholder groups.

The observed differences in knowledge of ostrich production and management (PC2) provide valuable insights into how familiarity with and understanding of farming practices vary across the category of participants, highlighting the influence of experience and exposure on knowledge levels. Farmers had the highest scores, stakeholders the intermediate, and consumers the lowest. This aligns with the expectation that those closer to the production process have greater knowledge, as also highlighted by Erian et al. (2019). In addition, gender, education, diet and province also influenced knowledge scores. Specifically, male participants, those without tertiary education, participants who preferred a meat diet, and participants from provinces with high ostrich production consistently gave higher scores. These patterns warrant further exploration. First, the finding that non-tertiary educated participants had higher knowledge scores supports the existing literature, which generally reports knowledge gaps and misconceptions about animal production systems (Clark et al. 2016). This lack of knowledge may be attributed to the fact that a small percentage of citizens are actively involved in the agricultural sector. The ostrich industry’s small scale and its confinement to specific regions in South Africa, such as the Western and Eastern Cape regions (Brand and Jordaan 2011; DALRRD, 2021), are probably additional contributing factors. These findings are consistent with those of Bir et al. (2019), who demonstrated that citizens in the United States, regardless of their involvement in the agricultural sector or level of tertiary education, have a limited understanding of food production processes. They also highlighted that a lack of knowledge is often associated with negative emotional attitudes towards livestock management and production practices. Social media and advertising can further exacerbate this incoherence (Rice et al. 2020). Consequently, misconceptions arising from a lack of understanding of husbandry practices in livestock industries may lead the public to accept that inadequate, potentially harmful techniques are commonly employed in intensive production systems. For instance, in poultry production, the public has raised concerns about slaughter practices, particularly inadequate stunning and slaughter without prior stunning (Erian and Phillips 2017). Second, responses from participants in provinces with higher ostrich production were associated with more knowledge, which is intuitive, as proximity to production may increase knowledge or exposure. For instance, Boogaard et al. (2006, 2011) demonstrated that lower proximity to, or limited exposure to, dairy farming systems was correlated with less knowledge/experience of dairy farming among Dutch and Norwegian participants. Third, these results may also reflect the characteristics of the sampled population, with a higher proportion of participants being men, directly involved in ostrich production, preferring a meat diet, residing in high ostrich-production provinces, and having less formal education.

The “attitude-behavior gap” was, however, evident in the willingness to pay for welfare-conscious products. Although welfare was highly valued, the willingness to pay a premium for welfare-conscious products was lower (mean ~ 3.61) compared to other welfare attitude scores. This observation aligns with similar research, where it was also reported that consumer willingness to pay for higher-welfare livestock products is generally positive but often marginal and varies by region and product type (Aschemann-Witzel and Zielke 2017). In addition, several studies highlighted that although most consumers reported paying attention to how animals were raised, and many were willing to pay a premium, their willingness to pay was mediated by ethical orientation. They also demanded certification/information for welfare-certified products (Giannetto et al. 2023; Miranda-de la Lama et al., 2017; Spain et al. 2018). A similar trend was observed in South Africa, where 76% of consumers expressed a willingness to pay more for certified animal welfare products (NSF, 2024). Compared to those figures, the lower willingness to pay in the present study may reflect specific constraints in the ostrich industry context (e.g., pricing, product awareness, niche market) or sample composition (e.g., stakeholders vs. general consumers). It may also reflect that other purchase criteria remain dominant (e.g., price, taste, convenience).

Although the data were collected prior to the COVID-19 pandemic, it provides an important baseline for understanding consumer perceptions of ostrich welfare. While the pandemic influenced food purchasing behavior and heightened concerns about food safety, farm animal welfare has remained an important factor in consumer attitudes and purchasing decisions (Meixner and Katt 2020; Naicker et al. 2021; Yang 2021). Therefore, although the prevalence of specific purchasing behaviors may have changed since data collection, the relationships between animal welfare perceptions and consumer intentions examined in this study remain relevant to livestock industries in general, and the ostrich industry in particular.

As such, these results have several actionable implications for the ostrich industry. The combination of modest self-reported knowledge of husbandry practices and strong welfare concern highlights a clear opportunity for the ostrich industry to engage in outreach and education. For instance, enhancing transparency and knowledge around ostrich production, welfare standards, and transport or slaughter procedures could strengthen trust and potentially increase willingness to pay a premium, as shown in other livestock sectors (Dickinson and Bailey 2005; Miranda-de la Lama et al., 2017; Xu et al., 2019). As participants place significant value on welfare and ethics, there is also an opportunity to develop local welfare certification or product labelling tailored to the ostrich industry (e.g., “Welfare Certified Ostrich”). Such initiatives could assist in reducing the gap between positive attitudes and actual purchasing decisions. However, the relatively low willingness to pay a premium suggests that producers and marketers should not assume that consumers will automatically spend more on welfare-conscious ostrich products. It may, however, increase the future and sustainability of the industry in light of changing global expectations and standards. Communicating welfare benefits alongside other desirable attributes (such as health benefits, origin, or sustainability) may increase market uptake (Li and Kallas 2021), particularly as the South African ostrich industry primarily serves European export markets (DALRRD, 2021). The demographic variation in knowledge and attitudes further suggests that different groups (e.g., based on gender, dietary habits, province of residence, or stakeholder category) may respond differently to welfare marketing messaging. For instance, women placed greater emphasis on welfare, while consumers in major production regions tended to score these priorities lower, potentially due to familiarity with industry practices. Tailored communication strategies may therefore be more effective than one-size-fits-all approaches (Córdoba et al. 2025). Finally, identifying underlying factors (such as welfare priorities, production knowledge, and health/ethical considerations) provides a useful framework for monitoring stakeholder attitudes over time. Future surveys can build on the identified factors to track shifts in perceptions and concerns within the ostrich industry.

It is essential to note that this study has several limitations. First, sampling was heavily weighted toward the high-ostrich-production province of the Western Cape (69.3%), and 81.3% of participants had a tertiary education qualification. This may limit generalisability to the broader South African public or to other provinces. Second, attitude measures may be subject to social desirability bias (participants may overstate welfare concerns). Third, willingness-to-pay measures are hypothetical and may not accurately reflect actual purchasing decisions (also referred to as the “hypothetical-bias” issue: Schmidt and Bijmolt 2020). As such, actual purchasing decisions (e.g., measurable premium payment, choice experiments) would strengthen the evidence base. Fourth, because the data were collected prior to the COVID-19 pandemic, they may not fully represent current consumer perceptions. However, these findings provide a valuable pre-pandemic baseline, particularly given evidence that the pandemic influenced consumer attitudes and behaviors (Sweeney et al. 2022). Future research could integrate choice modelling to examine the price premium consumers are willing to pay for welfare-conscious products. Fifth, the survey only captures snapshots of attitudes and knowledge at a single point in time. Longitudinal studies would help track how attitudes, knowledge and purchasing decisions evolve over time, especially if welfare education or marketing interventions are undertaken. Sixth, the ostrich industry has unique production, marketing, and consumer dynamics compared to mainstream poultry, pork, or beef production. Therefore, the findings may only partially align with other sectors. Future research might explore: (a) consumer choice experiments for ostrich-meat or ostrich products to measure actual willingness to pay under market conditions; (b) qualitative work (focus groups, interviews) with farmers, processors and consumers to understand barriers and enablers of welfare-conscious purchasing; (c) intervention studies to test whether welfare-labelling or informational campaigns increase willingness to pay and actual uptake in the sector; and (d) comparative studies across provinces or countries to examine whether geographic/production-density effects persist under a more balanced sampling population.

## Conclusions

Overall, this study provides insight into differences in viewpoints among South African participants regarding ostrich production, welfare, purchasing behavior, and husbandry preferences. Regarding knowledge of current production processes, participants demonstrated a limited to moderate understanding of ostrich farming practices, with knowledge varying by participant demographics and characteristics, and being greatest among individuals with direct involvement in the industry, such as farmers and stakeholders. In contrast, participants showed strong awareness of the welfare implications associated with ostrich production, with great importance placed on meeting fundamental welfare needs, reducing stress and pain, promoting natural behaviors, maintaining health, and ensuring competent stockmanship. Regarding purchasing behavior, animal welfare was recognized as an important consideration, and participants expressed a preference for welfare-conscious products; however, the lower willingness to pay a premium suggests that positive welfare perceptions may not always translate into purchasing decisions. Finally, participants demonstrated clear preferences for welfare-oriented ostrich husbandry practices, including appropriate handling, preventive healthcare, skilled stockperson management, and the implementation of formal welfare protocols. Collectively, these findings indicate that although knowledge of industry practices varies across participant categories, animal welfare is a central component of public expectations regarding ostrich production in South Africa, highlighting the importance of improved communication, transparency, and developing and adhering to welfare assurance strategies within the industry.

## Supplementary Information

Below is the link to the electronic supplementary material.

Supplementary Material 1

Supplementary Material 2

Supplementary Material 3

Supplementary Material 4

Supplementary Material 5

## Data Availability

All data of the questionnaire are available upon reasonable request.

## Declarations

### Generative AI

During the preparation of this work, the authors used the generative AI tool Grammarly to enhance readability. After utilizing this tool, the authors reviewed and edited the content and assumed full responsibility for its publication.
