# Supplementary material for: Perceptions of ostrich welfare and ethical practices: insights from consumers, farmers and stakeholders
Source: Trop Anim Health Prod. 2026 Oct 20;58(8):630. doi: 10.1007/s11250-026-05422-x (PMC13683527; doi:10.1007/s11250-026-05422-x)
Supplement: Supplementary file 1 [file 11250_2026_5422_MOESM1_ESM.docx]

**APPENDIX 1: QUESTIONNAIRE LAYOUT**

**SECTION A (Mark with an X)**

| **1** | **Age** | *<20* | *20-35* | *36-50* | *51-65* | *>65* |  |
| --- | --- | --- | --- | --- | --- | --- | --- |
| **2** | **Gender** | *Male* | *Female* | *Prefer not to say* | *Other* |  | |
| **3** | **Highest level of education** | *Unschooled* | *Primary school* | *Secondary/High*  *school* | *Tertiary (College/*  *University)* |  |  |
| **4** | **Occupation: please specify** |  | | | | | |
| **5** | **Income level** | *<R50 000 per*  *year* | *R50 000- R200*  *000 per year* | *R200 000-R400*  *000 per year* | *R400 000-R600*  *000 per year* | *>R600 000 per*  *year* | *Undisclosed* |
| **6** | **Country/Province of residence: please specify** |  | | | | | |
| **7** | **Dietary preference** | *No preference* | *Preference for*  *meat* | *Vegetarian* | *Vegan* | *Other (specify)* | |

**SECTION B (Mark with an X)**

| 1 | **How would you rate your understanding of the**  **following ostrich production and management procedures?** | *Poor/ No knowledge* | *Limited* | *Moderate* | *Knowledgeable* | *Very knowledgeable* |
| --- | --- | --- | --- | --- | --- | --- |
|  | General ostrich husbandry |  |  |  |  |  |
|  | Transportation of ostriches |  |  |  |  |  |
|  | Handling of ostriches |  |  |  |  |  |
|  | Artificial incubation of eggs |  |  |  |  |  |
|  | Human rearing of chicks |  |  |  |  |  |
| 2 | **How important do you consider the following**  **factors for the well-being of ostriches?** | *Not important* | *Less important* | *Neutral* | *Important* | *Very important* |
|  | Feed availability |  |  |  |  |  |
|  | Feed quality |  |  |  |  |  |
|  | Water availability |  |  |  |  |  |
|  | Water quality |  |  |  |  |  |
|  | Chick housing |  |  |  |  |  |
|  | Shelters in outside camps |  |  |  |  |  |
|  | Stocking density |  |  |  |  |  |
|  | Body condition of ostriches |  |  |  |  |  |
|  | Internal parasites |  |  |  |  |  |
|  | External parasites |  |  |  |  |  |
|  | Limited stress |  |  |  |  |  |
|  | Absence of pain |  |  |  |  |  |
|  | Ability to exhibit natural behaviour |  |  |  |  |  |
|  | Freedom of movement |  |  |  |  |  |
|  | Environmental enrichment |  |  |  |  |  |

| **3** | **How important do you consider the following**  **management and production practises for the well-being of ostriches?** | *Not important* | *Less important* | *Neutral* | *Important* | *Very important* |
| --- | --- | --- | --- | --- | --- | --- |
|  | Manually assisting chicks to hatch |  |  |  |  |  |
|  | Human rearing of chicks (as opposed to natural rearing  of chicks by breeding birds) |  |  |  |  |  |
|  | Frequent visual inspection of ostriches |  |  |  |  |  |
|  | Regular handling of ostriches |  |  |  |  |  |
|  | Stockmanship skill/experience |  |  |  |  |  |
|  | Type of vehicle/trailer used for transporting ostriches |  |  |  |  |  |
|  | Isolation of sick/injured birds |  |  |  |  |  |
|  | Preventative medication |  |  |  |  |  |
| **4** | **How important do you think it is that people**  **involved in ostrich production are knowledgeable with regards to the following?** | *Not important* | *Less important* | *Neutral* | *Important* | *Very important* |
|  | Anatomy of ostriches |  |  |  |  |  |
|  | Behaviour of ostriches |  |  |  |  |  |
|  | Detecting signs of stress in ostriches |  |  |  |  |  |
|  | Detecting signs of disease/illness in ostriches |  |  |  |  |  |
|  | Handling and restraining of ostriches |  |  |  |  |  |
|  | Transporting of ostriches |  |  |  |  |  |
| **5** | **How important do you consider the following?** | *Not important* | *Less important* | *Neutral* | *Important* | *Very important* |
|  | The welfare of farmed ostriches |  |  |  |  |  |
|  | The welfare of slaughter animals for product quality |  |  |  |  |  |
|  | Animal welfare when buying animal products |  |  |  |  |  |
|  | Implementing a formal welfare protocol for the  production of ostriches (meat, leather, feathers) |  |  |  |  |  |

| **6** | **How likely are you to:** | *Not likely* | *Less likely* | *Neutral* | *Likely* | *Very likely* |
| --- | --- | --- | --- | --- | --- | --- |
|  | Buy a product that originates from a welfare conscious  farm rather than a product from a welfare neutral farm? |  |  |  |  |  |
|  | Pay more for a product that originates from a welfare  conscious farm and production line? |  |  |  |  |  |
| **7** | **Any additional comments?** |  | | | | |
