# Supplementary material for: Perceptions of ostrich welfare and ethical practices: insights from consumers, farmers and stakeholders
Source: Trop Anim Health Prod. 2026 Oct 20;58(8):630. doi: 10.1007/s11250-026-05422-x (PMC13683527; doi:10.1007/s11250-026-05422-x)
Supplement: Supplementary file 2 [file 11250_2026_5422_MOESM2_ESM.docx]

**Supplementary Table S4. Comparison of eigenvalues and variance explained by the first three principal components (PC) for the imputed and complete-case datasets for the questionnaire about ostrich welfare perceptions**
