# Supplementary material for: Perceptions of ostrich welfare and ethical practices: insights from consumers, farmers and stakeholders
Source: Trop Anim Health Prod. 2026 Oct 20;58(8):630. doi: 10.1007/s11250-026-05422-x (PMC13683527; doi:10.1007/s11250-026-05422-x)
Supplement: Supplementary file 3 [file 11250_2026_5422_MOESM3_ESM.docx]

**Supplementary Table S1: Principal component (PC) loadings for all questionnaire items included in the principal component analysis of respondents' perceptions of ostrich production, management, welfare, and consumer behaviour.**
