# Supplementary material for: Perceptions of ostrich welfare and ethical practices: insights from consumers, farmers and stakeholders
Source: Trop Anim Health Prod. 2026 Oct 20;58(8):630. doi: 10.1007/s11250-026-05422-x (PMC13683527; doi:10.1007/s11250-026-05422-x)
Supplement: Supplementary file 4 [file 11250_2026_5422_MOESM4_ESM.docx]

**Supplementary Table S2: Missing data summary for questionnaire items, including the number and percentage of missing responses prior to imputation**
